# Service Quality and Related Factors in Primary Health Care Services: A Cross-Sectional Study

**DOI:** 10.3390/healthcare12100965

**Published:** 2024-05-08

**Authors:** Mehmet Sait Değer, Halim İşsever

**Affiliations:** 1 Department of Public Health, Medical Faculty, Hitit University, 19030 Corum, Türkiye; 2. Department of Public Health, Istanbul Medical Faculty, Istanbul University, 34093 Istanbul, Türkiye; hissever@istanbul.edu.tr

**Keywords:** SERVQUAL, perceived service quality, personality traits, primary health care services

## Abstract

Primary health care services aim to prevent diseases and improve health efficiently and effectively. This study measures perceived service quality in a primary healthcare organization and examines the effect of personality traits on service quality. The cross-sectional study population comprised individuals over the age of 18 who applied to the Bingöl Central Community Health Centre. A total of 460 participants were included in the study between November 2018 and March 2019. The participants completed a face-to-face questionnaire that included socio-demographic characteristics, the SERVQUAL Scale, and an abbreviated form of the revised Eysenck Personality Questionnaire. This study is based on doctoral research in public health. The study found median values for personality trait sub-dimensions as follows: neuroticism: 2, psychoticism: 2.65, extraversion: 4, and lying: 5. The SERVQUAL Score was −0.02. The study revealed that the quality of primary health care services did not meet the participants’ expectations. The study findings also indicated that age, educational attainment, and extraverted and psychotic personality traits were significantly associated with the satisfaction of service quality expectations (*p* < 0.05). It is recommended to provide primary health care services in facilities with good physical characteristics, with sufficient and competent health personnel, and in a timely and accurate manner to improve service quality.

## 1. Introduction

The concept of health is complex and dynamic, encompassing both objective criteria and subjective evaluations. These include the individual’s thoughts and perceptions about health. The multidimensional goal of the individual related to health is to prevent diseases, improve health, and reach a state of complete well-being in which the individual will lead a long life [1,2,3,4]. In this context, health services are provided on the basis of primary health care services with the objective of protecting the health of individuals and improving the health level of society by preventing diseases [3,5]. It is of paramount importance that primary health care services are accessible, effective, and efficient in order to cover the whole community [6,7,8]. Given that primary health care services represent the primary point of contact for individuals with the health system, they exert a significant influence on the health status of society as a whole and play a pivotal role in enhancing the health status of society and improving the quality of life of individuals [3,7,8,9].

The factors of population growth, prolonged life expectancy, and the diversification of health problems collectively increase the demand for health services. Furthermore, the dearth of health personnel to meet the demand for health services, which is a global phenomenon, renders access to health services challenging and affects the utilization of health services. Furthermore, economic changes, technological developments, individual expectations, and competitive conditions have elevated the significance of quality in health services [10,11,12,13]. These shifts and developments have begun to elevate the expectations of citizens from health services [14,15]. Conversely, health facilities that offer high-quality services will meet the expectations of individuals and enhance their satisfaction [10,11,16]. Satisfaction with primary health care services is influenced by a number of factors, including accessibility, communication, the quality of care provided, technical skills, and personal qualities [17,18]. A number of studies conducted within the scope of health services have demonstrated that digital health technologies and artificial intelligence applications are playing an increasingly important role in the delivery of health services [19]. These technologies facilitate the provision of innovative and continuous healthcare services by enhancing the practicality of qualified and accurate patient management, expeditious and high-quality service delivery, and effective communication [20,21,22]. From the perspective of citizens, the utilization of digital health technologies and artificial intelligence applications has the potential to enhance satisfaction with health services. This is achieved by facilitating access to health services, reducing costs, and personalizing the service [23].

A number of studies on healthcare service quality have demonstrated that healthcare facilities do not meet the expectations of citizens, with expectations being higher in the dimensions of reliability and enthusiasm. The manner in which citizens received the service they demanded on time, with respect and interest through competent and relevant health personnel, affected their satisfaction [24,25,26,27,28,29,30,31,32,33,34,35]. A number of factors have been identified as affecting the level of satisfaction with health services, including age, gender, marital status, income level, education level, length of hospitalization, and occupation [36,37,38,39,40,41]. Additionally, it has been postulated that personality characteristics may also influence the quality of the service provided. This is because personality traits can affect an individual’s ability to maintain healthy life behaviors, health perception, and well-being [42]. Individuals with different personality traits perceive health and illness at different levels of importance [43,44]. Personality traits affect the behaviors of the individual, which in turn affect the use of health services and satisfaction with these services [45].

In Turkey, primary healthcare services are provided through Community Health Centers (CHCs) and Family Health Centers (FHCs) [46,47,48]. Preventive health services are predominantly provided by different units, including Tuberculosis Control Dispensary (VSD) and Cancer Early Diagnosis, Screening and Education Center (KETEM) units within the CHC. In accordance with the parameters of the national cancer screening program, KETEMs are responsible for the implementation of screening programs for breast cancer (40–69 years) and cervical cancer (30–65 years) in women and colorectal cancer (over 50 years) in both women and men. The Tuberculosis Control Program is carried out in provinces through VSDs. Diagnostic examination, laboratory (sputum examination), and imaging (PA Lung X-ray) procedures on suspected patients are carried out in these units. Patients with a definitive diagnosis are provided with medication, and regular treatment follow-up is carried out using the direct supervised treatment (DGT) method [47,49].

It is important to evaluate the perceived service quality in these health facilities in order for primary health care services to be preferred, used at the desired level, and to have an impact on the quality of life of individuals. Therefore, it is crucial for health organizations to measure service quality in order to determine whether their services meet the needs of citizens and the functionality of their systems. This helps to identify and eliminate defects, improve the existing system, and strengthen the organizational structure. Consequently, health institutions can develop quality and continuous services that meet the needs of citizens and satisfy them [36,50,51,52]. This study aims to measure the service quality perceived by individuals applying to a primary healthcare organization and to examine the effect of socio-demographic and personality characteristics on service quality.

## 2. Materials and Methods

### 2.1. Study Design and Participants

This cross-sectional study was conducted in Bingöl, a province in eastern Turkey. The population of Bingöl, where the research was conducted, was 281,205 in 2018. In that year, 48.96% of the population was female, with 56.96% residing in the city center [53]. The Bingöl Central CHC KETEM and VSD units were selected for the study as they represent the primary centers where cancer screening programs and tuberculosis control programs are carried out in the province. The aim of the study was to evaluate the service quality of the health facilities and to investigate the related factors. Therefore, the population of the study consisted of people over the age of 18 who applied to the VSD and KETEM units of Bingöl Central CHC. A sample was selected from the population using a simple random sampling method to prevent loss of labor and time. In our research, the minimum number of participants to be reached by sample size analysis with a 95% confidence interval and 80% power was calculated as 392. However, based on our previous field research experiences, we considered the possibility that some participants might complete the questionnaire incompletely or incorrectly during the research process, potentially leading to their exclusion from the evaluation. With the prediction that the number of participants to be excluded from the evaluation would be 20% of the sample size, we determined the number of participants to be included in the research to be 470. Individuals aged 18 years and over who agreed to participate in the survey among those who applied to Bingöl Central CHC VSD and KETEM units were included in the study. The survey was conducted face to face with 472 participants between 1 November 2018 and 1 March 2019. Informed consent was obtained from all participants included in the study. Incorrect and incomplete forms were excluded from the evaluation, and a total of 460 forms were evaluated. This study was produced from a public health doctoral research.

### 2.2. Data Collection Tool and Measurements

In the study, a questionnaire consisting of three parts was applied face to face to those who applied to the Community Health Center. The entire questionnaire consists of a total of 73 questions.

### 2.3. Definitive Measurements

The first part of the questionnaire consists of five questions. In this section, participants were asked questions about their age, gender, marital status, education level, and place of residence.

### 2.4. Personality Trait Measurement (EPQR-A)

The abbreviated version of the revised Eysenck Personality Questionnaire (EPQR-A) was used to assess personality traits. The scale comprises 24 questions and includes sub-dimensions for psychoticism, neuroticism, extraversion, and lying. Extraverted personality traits include sociability, talkativeness, entrepreneurship, warmth, activity, and excitability. People with neurotic personality traits tend to exhibit attitudes and behaviors such as anxiety, fear, worry, emotionality, nervousness, irritability, sudden flashes of anger, depression, emotional inconsistency, and low self-esteem. People with psychotic personality traits are cold, aggressive, lacking in empathy, distant, selfish, insensitive, dreamy, and insecure. The Lying dimension defines people who try not to show their true personality and personality traits through their behavior. Respondents provide answers to the questions with a ‘yes’ or ‘no’ response format. Positive statements are scored as ‘yes’ (1 point) and ‘no’ (0 points), while negative statements undergo reverse scoring. Each sub-dimension of the scale comprises six statements, with a maximum score of 6 points for each sub-dimension. A high score indicates possession of more of those personality traits [54,55,56]. The scale’s Turkish validity and reliability study was conducted by Karancı et al. in 2007 [54].

### 2.5. Perceived Service Quality Measurement (SERVQUAL Scale)

The SERVQUAL Scale for Perceived Service Quality comprises 44 questions divided into two sections that assess expectations and perceptions of service quality using a 5-point Likert-type evaluation. The expectation section contains 22 questions that score the service expected from an ideal health facility. Similarly, the perception section contains the same 22 questions that participants use to evaluate and score the service they received from the health facility they visited. The SERVQUAL scale comprises five sub-dimensions: physical characteristics, reliability, enthusiasm, trust, and empathy. The physical characteristics sub-dimension comprises the initial four questions, which evaluate the physical condition of the service provider, the equipment used, and the appearance of the personnel. The reliability sub-dimension comprises questions 5–9, which evaluate the timely and accurate delivery of the promised service. The eagerness sub-dimension comprises questions 10–13, which evaluate the willingness of the staff to provide service and the fast delivery of the service. The trust sub-dimension comprises questions 14–17, which evaluate the employees’ knowledgeable, experienced, and polite behaviors and their ability to create trust. The empathy sub-dimension comprises questions 18–22, which evaluate the individual interests of the employees by paying attention to the demands of the person. Service quality is evaluated by calculating the difference between perceived and expected service, measured in points. The formula for perceived service quality is Perceived Service Quality = Perceived Service—Expected Service. The SERVQUAL Score (score) is calculated for each question, and the average score of the questions in the sub-dimensions gives the sub-dimension score. The average of the sub-dimension scores expresses the average SERVQUAL Score, which can range from +4 to −4 [57,58,59]. A SERVQUAL Score of + indicates that expectations have been exceeded, while a score of − (negative) indicates that expectations have not been met. A score of 0 indicates that expectations have been minimally met.

### 2.6. Statistical Analysis

The descriptive data were presented as percentages and either mean ± standard deviation (SD) or median (min–max). To evaluate whether the data were normally distributed, the Kolmogorov–Smirnov and Shapiro–Wilk tests were used. As the data obtained from the participants were not normally distributed, nonparametric tests were used. For our study, we used the Mann–Whitney U Test to compare two groups and the Kruskal–Wallis test to compare more than two groups, as the data of the SERVQUAL Scale and the EPQR-A were not normally distributed. We accepted the alpha value as 5% and the confidence interval as 95% and considered statistical significance as *p* < 0.05. The study used linear regression analysis to predict perceived service quality according to a number of independent variables, such as age, education level, and personality traits. The research data were analyzed using Statistical Package for the Social Sciences (SPSS) 22.0.

### 2.7. Ethic Decision

Ethics Committee approval (number 1284, date 20 September 2018) was obtained from Istanbul University Istanbul Faculty of Medicine Clinical Research Ethics Committee, and institutional permission (number 81966737-044, date 12 October 2018) was obtained from Bingöl Provincial Health Directorate.

## 3. Results

The research group had an average age of 48.21 ± 11.75 years, with the youngest participant aged 18 years and the oldest aged 76 years. The socio-demographic characteristics of the research group are shown in Table 1.

The equal-weighted scores and sub-dimension scores of the research participants on the SERVQUAL Scale are shown in Table 2.

Women scored significantly higher than men in the trust sub-dimension, and married individuals scored significantly higher than single individuals. In the sub-dimension of physical characteristics, individuals with primary school education or below scored significantly higher than those with undergraduate or higher education. Table 3 presents the distribution of SERVQUAL Scale and sub-dimension scores based on socio-demographic characteristics in the research group.

Table 4 displays the median (min–max) values of EPQR-A sub-dimensions.

Table 5 shows the distribution of EPQR-A and sub-dimension scores based on socio-demographic characteristics in the research group. Statistically significant differences were observed in the extraversion sub-dimension between the 18–30 age group and other age groups, as well as in the neuroticism and psychoticism sub-dimensions between the 18–30 age group and the over-40 age group. Additionally, significant differences were found in the lying sub-dimension between the 31–40 age group and the over-40 age group. In the sub-dimensions of neuroticism and psychoticism, women scored significantly higher than men. Additionally, in the psychoticism sub-dimension, single people scored higher than married people (*p* = 0.007).

It was observed that individuals with less than a primary school education scored lower in the sub-dimensions of extraversion and neuroticism than those with a high school education (*p* = 0.001). The statistical difference in the sub-dimension of lying is a result of pairwise comparisons between different educational levels. Specifically, there is a significant difference between the high school and college and above groups (*p* = 0.017), as well as between the primary school and below and college and above groups (*p* = 0.010).

Table 6 presents the results of a linear regression analysis of perceived service quality according to independent variables. The analysis revealed that extraversion and psychoticism, which are sub-dimensions of EPQR-A, and age and education level variables contributed significantly to the model. As extraversion and psychoticism personality traits become more prominent, the probability of perceived service quality meeting expectations decreases. Similarly, as age and education level increase, the probability of service quality meeting expectations decreases.

## 4. Discussion

The mean age of the study group was 48.21 ± 11.75 years, which is higher than both the national average (33.44) and the provincial average (29.74) [53]. Almost half of the participants (48.2%) were over 50 years of age. The relatively high average age of the participants in our study can be attributed to the fact that they were individuals who had applied to cancer screening programs.

Personality is formed by a combination of innate characteristics, experiences, emotions, and thoughts acquired later in life. It affects the attitudes, behaviors, and expectations of the individual. A number of factors, including socio-demographic characteristics (age, gender, education level, occupation, working conditions, place of residence) and physical and psychosocial well-being, may influence an individual’s perception of health and personality characteristics [60,61,62,63,64,65,66]. Studies conducted with over 40,000 participants in the USA, Europe, and Japan have demonstrated that extraversion is associated with a positive perception of health, while neuroticism is associated with a negative perception of health [44]. It is important to note that health perception and personality traits may mutually influence each other. Furthermore, individuals with positive health perceptions exhibit more social, active, and emotionally consistent behaviors [67,68]. It is also important to note that personality may also affect an individual’s well-being and life satisfaction [69]. In our study, we found that personality traits were associated with gender, age, marital status, and education level. Furthermore, the scores for psychoticism and lying were found to be higher in our study than in previous studies, while the scores for extraversion and neuroticism were found to be lower [54,70].

Personality traits are the result of an individual’s genetic makeup, environmental factors, and life choices. They can have a significant impact on a person’s health. Individuals who exhibit sociable, communicative, talkative, sensitive, and emotionally coherent traits tend to have better cognitive and social well-being. Adopting healthy lifestyle habits can help maintain and improve physical health for people with these personality traits. However, individuals who exhibit introverted, asocial, anxious, and insensitive behaviors and who react impulsively may experience poor psychosocial well-being. This may have a negative impact on their health as well as their expectations and perceptions of health services [71]. The results of our study indicate that extraversion (β: −0.128, t: −2.554, *p*: 0.011) and psychotic (β: −0.112, t: −2.175, *p*: 0.030) personality traits contribute to dissatisfaction with service quality. In other words, individuals with talkative, social, and active personality traits and individuals with asocial, insensitive, and selfish attitudes and behaviors were found to have higher expectations from health services. This situation seems to have significantly affected their dissatisfaction with health services.

In our research group, the highest expectation in terms of service quality came from the trust sub-dimension, while the lowest expectation came from the physical features sub-dimension. In the literature, the highest expectation is generally found in the reliability sub-dimension, while the lowest expectation is found in the physical features sub-dimension. However, in our study, it was observed that the highest perception was in the trust sub-dimension, while the lowest perception was in the physical features sub-dimension. The research group participants expressed the lowest level of satisfaction with the physical appearance of the health facility during the process of receiving health services. Although the respondents had low expectations regarding the physical characteristics of the health facility, the greatest expression differences were observed in this sub-dimension. This indicates that the physical appearance and equipment of Bingöl Central CHC VSD and KETEM units are inadequate. Their visual attractiveness is weak, and they fail to meet the service expectations of citizens.

The level of satisfaction of individuals with health services affects their choice of health institution [51,72]. Socio-demographic factors such as age, gender, education level, income level, and occupation are associated with service quality and significantly affect individuals’ choice of health institution [23,24,50,73,74,75,76,77]. According to studies, young, highly educated, and high-income individuals perceive lower levels of service quality [59]. Conversely, elderly, low-educated, and low-income individuals have lower expectations of health services but perceive service quality at a higher level [20,22]. In our study, educational level, marital status, and gender were found to be related to perceived service quality. The study revealed that women exhibited greater confidence in the knowledgeable attitudes and polite behaviors of the VSD and KETEM staff compared to men (*p* = 0.048) and married people compared to single people (*p* = 0.020). Participants with lower educational levels were more satisfied with the physical characteristics of the VSD and KETEM units (*p* = 0.010). As the level of education increased, satisfaction with the physical characteristics of health facilities decreased. Furthermore, age (β: −0.137, t: −2.535, *p*: 0.012) and educational level (β: −0.143, t: −2.780, *p*: 0.006) were found to be significant predictors of satisfaction with service quality. As age and educational level increase, the probability of service quality meeting expectations decreases.

The research group found the Equal-Weighted SERVQUAL Score to be −0.02. This score indicates that the expectations of applicants to Bingöl Central CHC VSD and KETEM units are not met. This situation is probably due to the outdated and unattractive structure of VSD and KETEM units. In fact, citizens perceived higher service quality in the SERVQUAL Scale compared to their expectations in sub-dimensions other than physical characteristics. Nevertheless, the low score achieved in the physical characteristics sub-dimension led to a low Equal-Weighted SERVQUAL Score, which represents the average of all sub-dimension scores. Consequently, the service quality perceived by the participants was below their expectations.

It is expected that healthcare providers will provide their services in a timely, accurate, and high-quality manner, as promised. In addition, the availability of appropriate services at a low cost, effective channels, and ease of access to services are crucial for the delivery of quality health services. This situation has a positive effect on individual perception and satisfaction, thus increasing the service [57,78,79,80]. Consequently, when receiving healthcare, both the qualitative and quantitative aspects of care in health institutions continue to be of paramount importance. However, individuals are less satisfied with quantitative (physical) changes [81,82,83]. Nevertheless, in our study, although they did not emphasize the importance of physical activity, the worsening of the physical change in the healthcare services provided by the service led to the perception of low service quality. This situation serves to highlight the necessity for an increase in the structure and hardware compatibility of healthcare facilities and the service standard. Nevertheless, contemporary times have witnessed a notable increase in the utilization of digital health applications. Consequently, individuals can receive more convenient, cost-effective, and expedient services remotely, rather than having to visit a health facility. The use of digital applications and methods in health services is of great importance, as they enable disabled and elderly individuals who have difficulty accessing health services to do so and to benefit from them [18,84].

## 5. Strengths and Limitations

Our study investigates the impact of personality traits on service quality in primary care and contributes significantly to the literature by examining the factors affecting service quality in the service recipient dimension. However, caution is necessary when generalizing the results to all primary health care services due to the study being conducted in a single center.

## 6. Conclusions

The service quality perceived by individuals who applied to Bingöl Central CHC KETEM and VSD units did not meet their expectations, as indicated by the SERVQUAL Score of −0.02. The perceived service quality was found to be associated with marital status (*p* < 0.05), gender (*p* < 0.05), and education level (*p* < 0.05). The median values of the participants in the extraversion (4) and neuroticism (2) sub-dimensions were lower than those reported in the literature, while the median values of the psychoticism (2.65) and lying (5) sub-dimensions were higher. The study found significant correlations between personality traits and age (*p* < 0.05), gender (*p* < 0.01), education level (*p* < 0.05), and marital status (*p* < 0.05). Moreover, the findings suggested that extroverted and psychotic personality traits, along with age and educational level, influenced the degree to which service quality met expectations (*p* < 0.05).

In healthcare settings, it is crucial that experienced, interested, and willing employees provide the services that citizens require accurately and promptly. In terms of service quality, it is of paramount importance that health facilities possess new equipment, keep them clean, and are aesthetically pleasing. Therefore, primary health care services in our country should be provided in facilities suitable for current conditions. The widespread use of digital health applications and artificial intelligence in healthcare services can enhance the satisfaction of individuals by facilitating the delivery of expedient, individualized, and qualified services.

Personality traits play a determining role in individual perceptions and evaluations of health and illness concepts. It is crucial to take into account personality traits during the implementation and evaluation of health services, as individuals with diverse personality traits may perceive health differently and consequently engage in distinct health-related decisions and behaviors.

## Figures and Tables

**Table 1:** Socio-demographic characteristics.

		n	%
Gender	Female	236	51.3
	Male	224	48.3
Age Group	18–30 Years	30	6.5
	31–40 Years	104	22.6
	41–50 Years	104	22.6
	51–60 Years	146	31.7
	>60 Years	76	16.5
Marital Status	Married	390	84.8
	Single	40	8.7
	Divorced/Widowed	30	6.5
Education Status	Primary school and below	239	52.0
	Middle school	52	11.3
	High school	84	18.3
	High school above	85	18.5
Place of Residence	Province	387	84.1
	District	40	8.7
	Town/Village	33	7.2

**Table 2:** SERVQUAL scale and subscale scores.

SERVQUAL Subscale	Score
**Tangibles**	−0.200
**Reliability**	0.025
**Responsiveness**	0.015
**Assurance**	0.040
**Empathy**	0.015
**Equal-Weighted SERVQUAL Score**	**−0.020**

**Table 3:** Comparison of SERVQUAL and subscale scores according to socio-demographic characteristics.

Gender		Tangibles	Reliability	Responsiveness	Assurance	Empathy	SERVQUAL Score
Female	**Mean Rank**	229.57	239.44	240.07	241.86 ^1^	240.08	234.52
Male		231.48	221.08	220.42	218.53 ^2^	220.41	226.27
	**MWU**	26.213	24.321	24.174	23.750	24.172	25.483
	***p***	0.873	0.122	0.096	0.048 ^1−2^	0.097	0.500
**Marital Status**							
Married	**Mean Rank**	233.10	231.16	232.24	236.67 ^1^	231.97	233.90
Single		230.38	230.48	213.99	179.14 ^2^	227.65	219.33
Divorced		196.83	222.02	229.93	218.78	215.25	201.23
	**KWH**	2.241	0.144	0.755	7.805	0.506	2.047
	***p***	0.326	0.931	0.686	0.020 ^1−2^	0.777	0.359
**Education Status**							
Primary school and below	**Mean Rank**	243.97 ^1^	232.19	237.43	240.70	236.94	239.54
Middle school		237.17	236.45	219.17	230.13	220.99	244.89
High school		229.32	239.65	223.54	224.52	233.45	219.00
High school above		189.71 ^2^	213.06	224.82	207.96	215.29	207.64
	**KWH**	11.453	2.184	1.559	4.454	2.176	4.992
	***p***	0.010 ^1−2^	0.535	0.669	0.216	0.537	0.165

*p*: Significance level, ^1,2^: Statements of groups with significant differences between them, MWU: Mann–Whitney U test, KWH: Kruskal–Wallis H test.

**Table 4:** EPQR-A and sub-dimension scores.

	Mean ± Sd	Median	Min–Max
**Extraversion**	3.54 ± 1.29	4	0–6
**Neuroticism**	2.57 ± 1.94	2	0–6
**Psychoticism**	2.61 ± 0.83	2.65	1–6
**Lying**	4.43 ± 1.30	5	0–6

Sd: Standard Deviation, Min: Minimum, Max: Maximum.

**Table 5:** Comparison of EPQR-A sub-dimension scores according to socio-demographic characteristics.

Age Group		Extraversion	Neuroticism	Psychoticism	Lying
18–30 Year	**Mean Rank**	322.03 ^1^	308.03 ^1^	307.43 ^1^	209.92
31–40 Year		240.42 ^2^	263.57	248.17	272.30 ^1^
41–50 Year		227.86 ^2^	231.02 ^2^	230.24 ^2^	216.27 ^2^
51–60 Year		226.58 ^2^	215.20 ^2^	210.05 ^2^	217.89 ^2^
>60 Years		191.95 ^2^	183.31 ^2^	215.59 ^2^	225.13
	**KWH**	22.740	28.854	18.953	16.483
	***p***	<0.001 ^1−2^	<0.001 ^1−2^	0.001 ^1−2^	0.002 ^1−2^
**Gender**					
Female	**Mean Rank**	232.64	251.71 ^1^	246.51 ^1^	239.60
Male		228.25	208.15 ^2^	213.63 ^2^	220.91
	**MWU**	25.927	21.426	22.652	24.283
	***p***	0.715	<0.001 ^1−2^	0.004 ^1−2^	0.097
**Marital Status**					
Married	**Mean Rank**	231.64	226.60	223.55 ^1^	233.11
Single		240.98	269.21	286.49 ^2^	225.23
Divorced		201.77	229.60	246.25	203.58
	**KWH**	1.787	3.822	9.982	1.745
	***p***	0.409	0.148	0.007 ^1−2^	0.418
**Education Status**					
Primary school and below	**Mean Rank**	210.22 ^1^	212.77 ^1^	232.82	221.92 ^1^
Middle school		259.81	247.17	213.91	232.05
High school		229.82	245.17	225.43	214.23 ^2^
High school above		270.28 ^2^	255.65 ^2^	239.14	269.75 ^3^
	**KWH**	16.719	9.360	1.585	11.692
	***p***	0.001 ^1−2^	0.025 ^1−2^	0.663	0.009 ^1−3,2−3^

*p*: Significance level, ^1,2,3^: Statements of groups with significant differences between them, MWU: Mann–Whitney U test, KWH: Kruskal–Wallis H test.

**Table 6:** Linear regression analysis of perceived service quality according to independent variables.

	B	Std. Error	β	t	Sig.	95% Confidence Interval		Collinearity Statistics	
						Lower	Upper	Tolerance	VIF
(Constant)	0.821	0.235		3.502	0.001	0.360	1.282		
Extraversion	−0.051	0.020	−0.128	−2.554	0.011	−0.091	−0.012	0.921	1.086
Neuroticism	−0.007	0.016	−0.026	−0.460	0.645	−0.038	0.023	0.704	1.421
Psychoticism	−0.069	0.032	−0.112	−2.175	0.030	−0.132	−0.007	0.870	1.150
Lying	−0.015	0.022	−0.038	−0.684	0.494	−0.059	0.028	0.737	1.357
SMEAN (Age)	−0.006	0.002	−0.137	−2.535	0.012	−0.011	−0.001	0.791	1.264
Education Level	−0.062	0.022	−0.143	−2.780	0.006	−0.105	−0.018	0.873	1.146

R: 0.226, R^2^: 0.051, Adjusted R^2^: 0.035, F: 3.675, *p*: 0.001, Durbin–Watson: 0.098.

## Data Availability

All datasets and analyses used throughout the study are available from the corresponding author upon reasonable request.
